# Genomic Sequence Analysis of Methicillin- and Carbapenem-Resistant Bacteria Isolated from Raw Sewage

**DOI:** 10.1128/spectrum.00128-21

**Published:** 2021-06-16

**Authors:** Mo Kaze, Lauren Brooks, Mark Sistrom

**Affiliations:** a Department of Life and Environmental Sciences, University of California, Merced, California, USA; b Utah Valley University, Orem, Utah, USA; University of Minnesota

**Keywords:** microbial genomics, antibiotic resistance, resistome, ecological microbiology

## Abstract

Antibiotic resistance is one of the largest threats facing global health. Wastewater treatment plants are well-known hot spots for interaction between diverse bacteria, genetic exchange, and antibiotic resistance. Nonpathogenic bacteria theoretically act as reservoirs of antibiotic resistance subsequently transferring antibiotic resistance genes to pathogens, indicating that evolutionary processes occur outside clinical settings and may drive patterns of drug-resistant infections. We isolated and sequenced 100 bacterial strains from five wastewater treatment plants to analyze regional dynamics of antibiotic resistance in the California Central Valley. The results demonstrate the presence of a wide diversity of pathogenic and nonpathogenic bacteria, with an arithmetic mean of 5.1 resistance genes per isolate. Forty-three percent of resistance genes were located on plasmids, suggesting that large levels of gene transfer between bacteria that otherwise may not co-occur are facilitated by wastewater treatment. One of the strains detected was a *Bacillus* carrying pX01 and pX02 anthrax-like plasmids and multiple drug resistance genes. A correlation between resistance genes and taxonomy indicates that taxon-specific evolutionary studies may be useful in determining and predicting patterns of antibiotic resistance. Conversely, a lack of geographic correlation may indicate that landscape genetic studies to understand the spread of antibiotic resistance genes should be carried out at broader scales. This large data set provides insights into how pathogenic and nonpathogenic bacteria interact in wastewater environments and the resistance genes which may be horizontally transferred between them. This can help in determining the mechanisms leading to the increasing prevalence of drug-resistant infections observed in clinical settings.

**IMPORTANCE** The reasons for the increasing prevalence of antibiotic-resistant infections are complex and associated with myriad clinical and environmental processes. Wastewater treatment plants operate as nexuses of bacterial interaction and are known hot spots for genetic exchange between bacteria, including antibiotic resistance genes. We isolated and sequenced 100 drug-resistant bacteria from five wastewater treatment plants in California’s Central Valley, characterizing widespread gene sharing between pathogens and nonpathogens. We identified a novel, multiresistant *Bacillus* carrying anthrax-like plasmids. This empirical study supports the likelihood of evolutionary and population processes in the broader environment affecting the prevalence of clinical drug-resistant infections and identifies several taxa that may operate as reservoirs and vectors of antibiotic resistance genes.

## INTRODUCTION

Antibiotic resistance (AR) is prevalent in bacterial populations occurring in both natural (1–3) and anthropogenically altered (4–6) environments. Many antimicrobial compounds used therapeutically occur naturally (7, 8), and subsequent mechanisms of resistance to them evolved long before their use as therapeutic agents to treat bacterial infections (9). It is expected that bacteria in the environment will carry antibiotic resistance genes (10); however, anthropogenic activity, including the overuse of antibiotic compounds in therapeutic and agricultural activities, has profound impacts on the evolution, geographic, and taxonomic distribution of antibiotic resistance (11–13). Understandably, most antibiotic resistance research focuses on clinically relevant species and strains of bacteria, yet the widespread use of antibiotics outside clinical settings means that important evolutionary and population processes occur in bacterial species and communities that are not considered pathogenic and clinically relevant (14, 15). These resistance mechanisms, leading to antibiotic resistance or loss of susceptibility to antibiotics and novel pathogenicity, may be transferred to clinically relevant pathogens through horizontal transfer and may be difficult to predict and combat (16, 17).

Understanding the spatial diversity of resistant organisms, genes, and phenotypes can allow for the extrapolation of antibiotic resistance beyond the biological and geographic systems in which they originate (18–20). The regional scale at which bacterial community resistance profiles vary is important in determining appropriate strategies to predict and combat antibiotic resistance at the local, county, and city scales (15, 21, 22). Resolving which bacterial taxa, both pathogenic and otherwise, harbor genes encoding antibiotic resistance, and the genomic context of these genes (e.g., chromosomal versus plasmid encoded), is foundational to determining the likelihood of transmission from a given environment (23). Wastewater treatment plants have been observed as hot spots for antibiotic resistance (24, 25). A confluence of bacteria from multiple sources, including runoff from domestic, clinical, and agricultural environments, facilitates gene transfer between bacteria from diverse environments and taxonomic backgrounds (26, 27). Regional differences between bacterial communities in wastewater treatment facilities potentially represent distinct risks and indicators of broader resistomes associated with particular geographic regions and human population centers.

The Central Valley of California is home to nearly 10 million people and has 12 major metropolitan centers (28). It is the most productive agricultural region in the United States, producing over $45 billion in agricultural sales annually (29). Significant agricultural industries in the Central Valley include dairy and beef feedlots, poultry, and pork production—all of which are significant users of agricultural antibiotics (30–32). Up to 80% of the antibiotics sold in the United States are used in agricultural rather than medical contexts (33), and approximately 70% of these are considered medically important (33). It has been suggested that antibiotic misuse in animal production is a substantial driver of antibiotic resistance (34–36). The Central Valley is a major, global nexus for the interaction of urban and agricultural microbial communities and therefore an area of particular interest and concern for the dissemination of resistance between disparate environments, and it may act as an informative model for the study of reservoirs and vectors of antimicrobial resistance.

**FIG 1:**
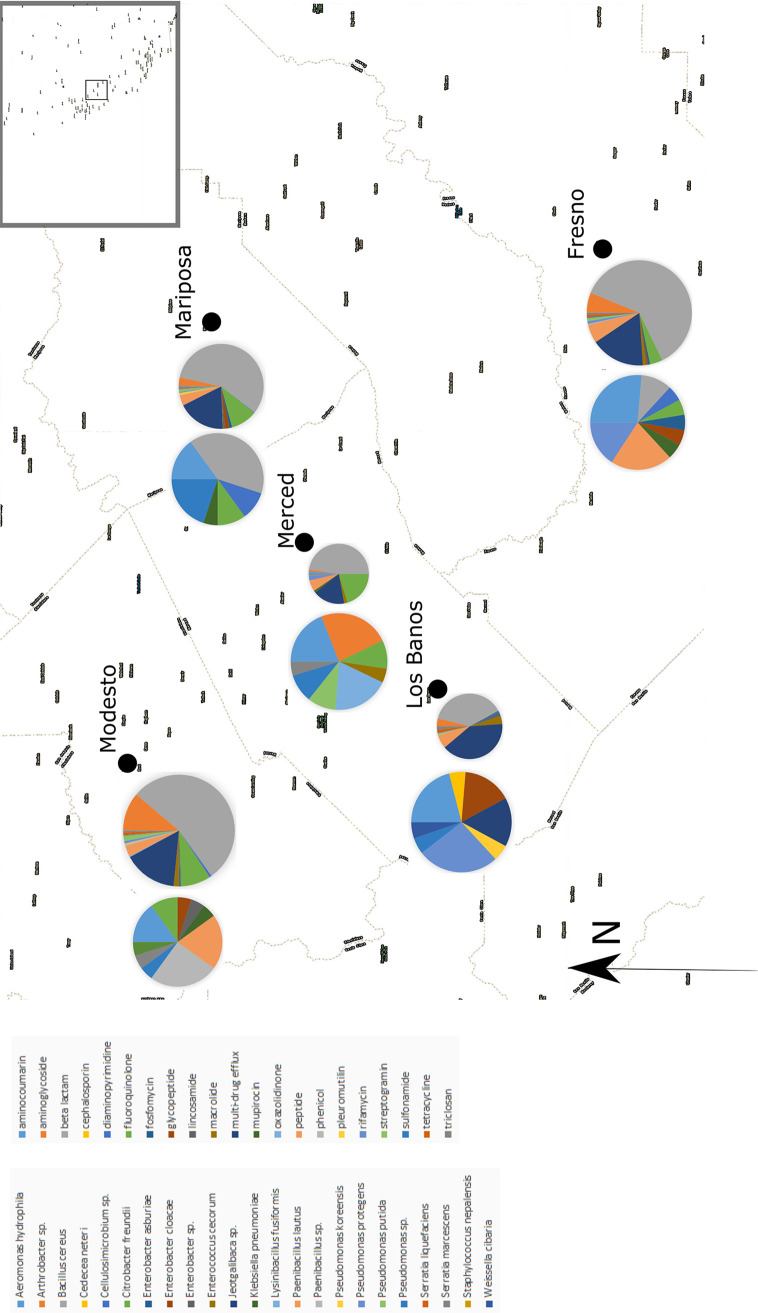
A map of the five sampling localities in the Central Valley of California. The pie chart on the left (labeled S) indicates species diversity of the 20 samples sequenced at each locality, and the pie chart on the right (labeled G) displays the drug classes of unique antibiotic resistance genes detected at each site, with each pie being proportional to the number of unique resistance genes detected at each site.

In this study, we sampled influent from five wastewater treatment plants across the Central Valley (Fig. 1). We cultured 10 methicillin-resistant and 10 carbapenem-resistant isolates from each locality and used whole-genome sequencing to analyze their genomic contents. We found a wide range of both pathogenic and nonpathogenic antibiotic-resistant bacteria across all sites, with no correlation between geographic distance and either species composition or resistance profile. We identified a correlation between resistance profile and species composition at a given site. These data and their characteristics allows for comparison with clinical data that can provide context for patterns of clinical infection and data useful for the mitigation of clinically relevant environmental antibiotic resistance.

## RESULTS

**FIG 2:**
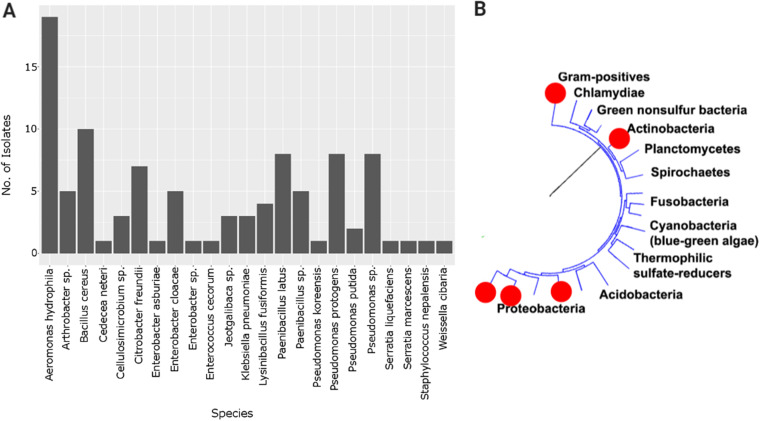
(A) Frequency histogram of species identifications of the 100 bacterial genomes sequenced. All samples were identified to genus level, and 75 were able to be identified to species level. The data set contains 16 genera and 18 species of bacteria. (B) Proximate phylogenetic placement of bacterial strains sequenced in the present study. Three phyla are represented in our data set: *Gammaproteobacteria*, *Actinobacteria*, and *Firmicutes*. (Tree adapted from reference 104.)

Across all sites we isolated a total of 16 genera of bacteria, with an arithmetic mean of 8.4 (standard deviation [SD], 1.62) per site (Fig. 2). Of the 100 isolates sequenced, 25 could not be identified beyond the genus level, and the remaining 75 isolates comprised 18 species.

**FIG 3:**
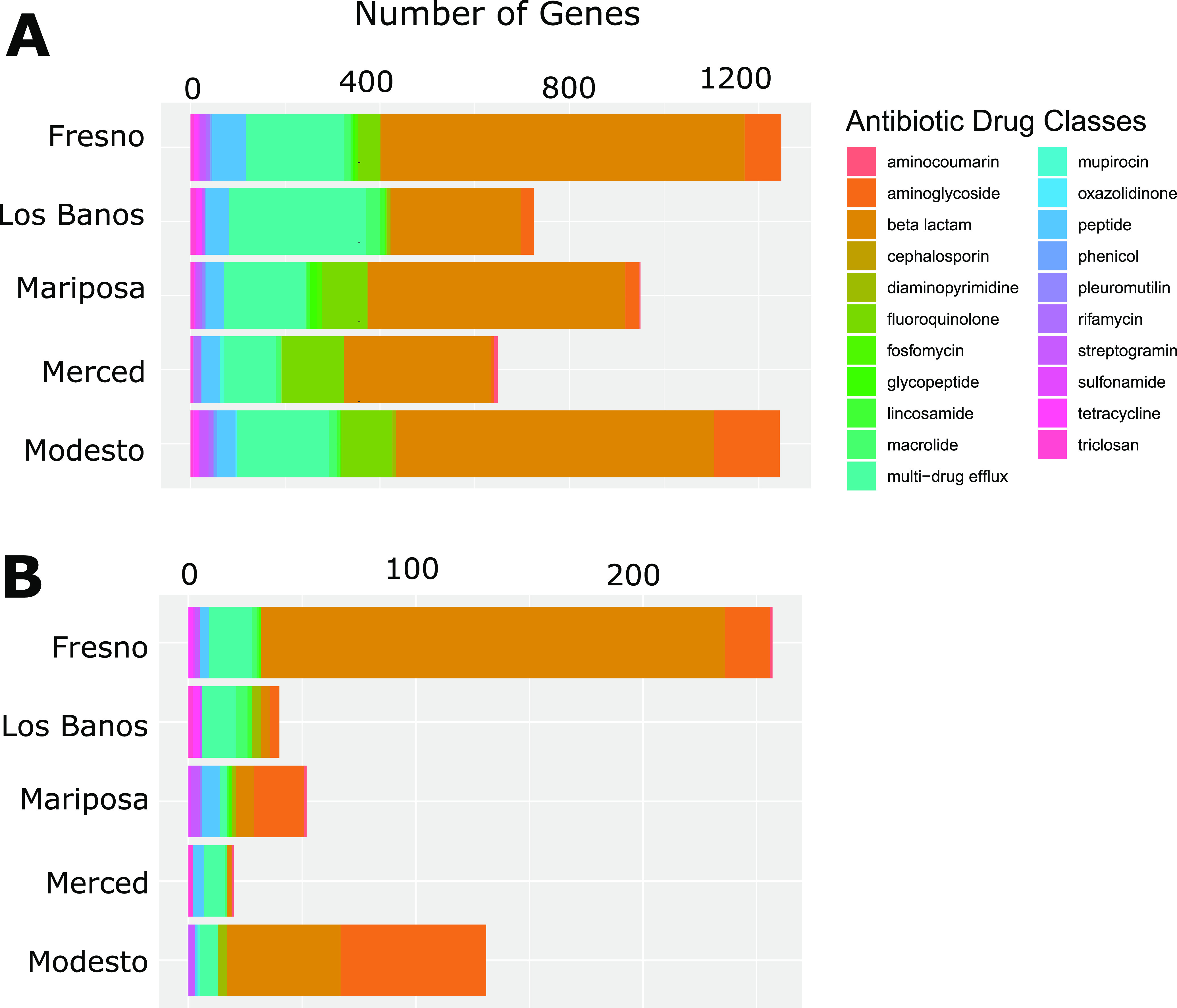
Bar plot of resistance genes detected at each site. (A) Total number of genes detected per drug class at each site. (B) Number of genes detected per drug class when restricted to those found on plasmids. The proportion of genes per site and drug class remain similar for total and putatively plasmid-borne resistance genes.

**FIG 4:**
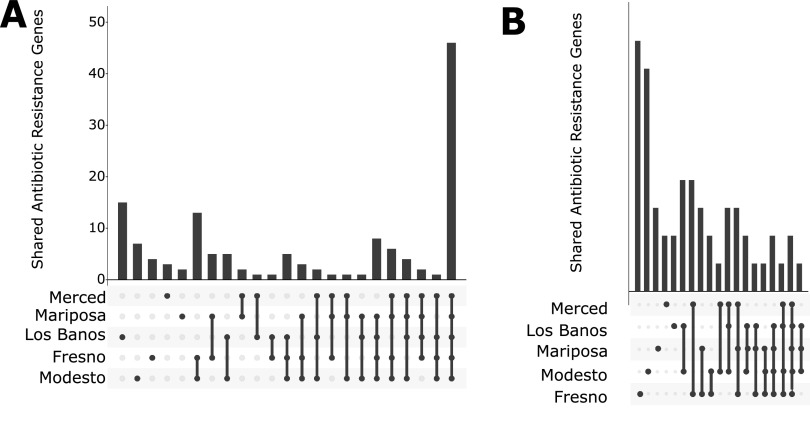
Gene co-occurrence analysis of AR genes by site. (A) Vertical bars indicate number of genes shared for each location; dots and connecting bars indicate the sites included in each group. (B) Intersection plot of genes found in each species when restricted to plasmid hits. Vertical bars indicate number of genes shared for each site; dots and connecting bars indicate the sites included in each group.

**FIG 5:**
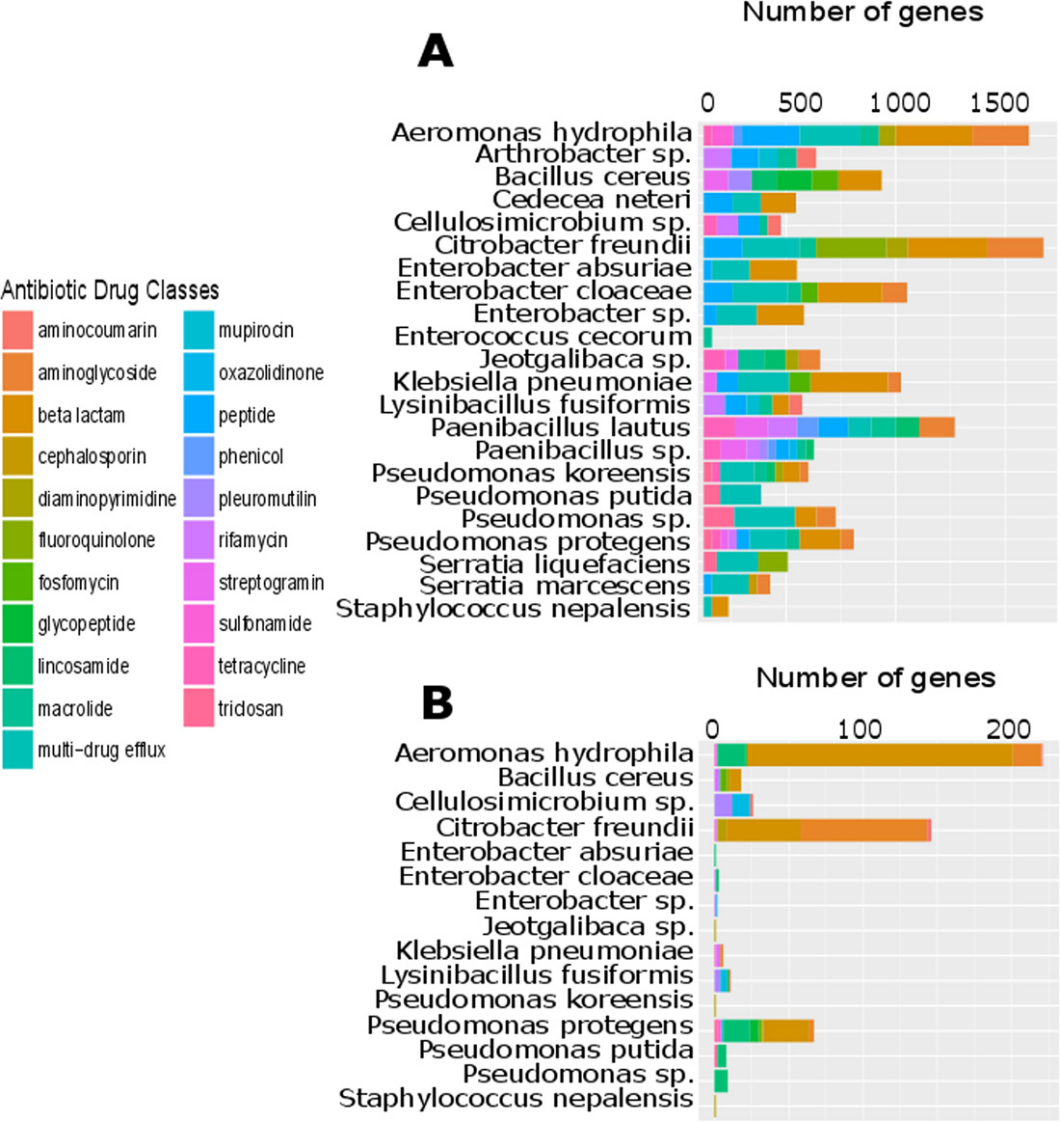
Bar plot of the resistome visualized by species. Most resistance genes are species specific; however, this trend is considerably more pronounced in genes carried on plasmids. (A) Number of genes detected per drug class for each species. (B) Number of genes detected per drug class when restricted to those found on plasmids.

**FIG 6:**
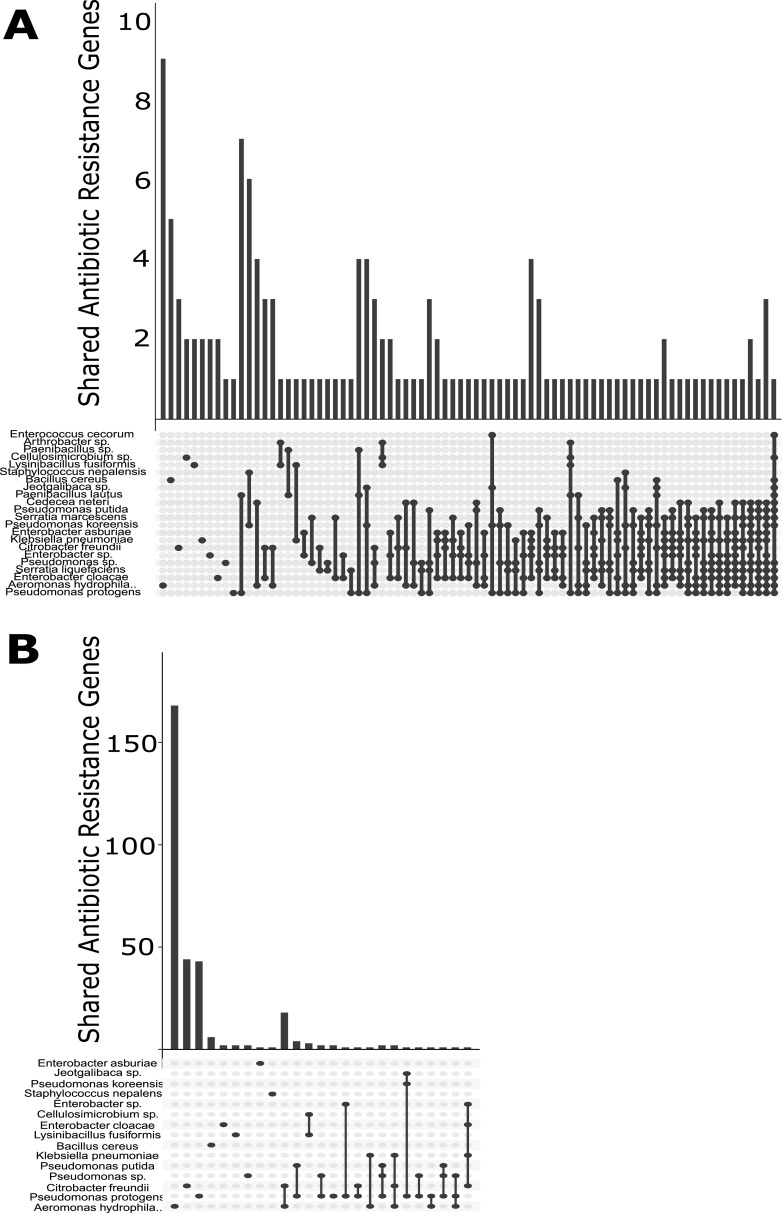
Gene co-occurrence analysis of AR genes by species. (A) Vertical bars indicate the number of genes shared for each species; dots and connecting bars indicate the species included in each group. (B) Intersection plot of genes found in each species when restricted to plasmid hits. Vertical bars indicate the number of genes shared for each species group; dots and connecting bars indicate the species included in each group.

All but two isolates—one *Paenibacillus* sp. and one Weissella cibaria isolate—had at least 2 known antibiotic resistance (AR) genes, with an arithmetic mean of 56.83 (SD, 70.62) gene hits per isolate. The arithmetic mean number of drug classes to which a given sample contained resistance genes was 5.1 (SD, 2.12). When visualized by site (Fig. 3 and 4) and species (Fig. 5 and 6), 22.1% of AR genes were specific to a single site, 26.4% were specific to a single genus, 32.9% were found in all sites, and none were found in all species.

A substantial proportion (43.4%) of isolates had resistance genes on plasmids, with an arithmetic mean of 11.30 (SD, 27.74) hits per isolate, when considering only isolates with AR genes on plasmids. The arithmetic mean number of drug classes a given isolate contained resistance genes to was 2.5 (SD, 1.58). As visualized by site (Fig. 3 and 4) and species (Fig. 5 and 6), 85.6% of AR genes were specific to a single site, 90.1% were specific to a single species, only a single gene (MexI) was found at all sites, and none were found in all species. No AR genes were found among predicted prophage genes.

For *Bacillus* species, all isolates were determined to carry Bacillus cereus toxin proteins (37, 38), and none contained B. thuringiensis diagnostic *cry* proteins (39). While no samples had both B. anthracis pX01 and pX02 plasmids (40), one sample (Fresno16) did contain pX01-like and pX02-like plasmids and was identified as B. cereus. Alignment to B. anthracis plasmids pX01 and pX02 yielded 98.9% and 98.0% length matches, with 1,142× and 1,492× coverages, respectively. However, pairwise identity with the reference sequences were 61.7% and 62.4%, respectively. Alignment to anthrax-like Bacillus cereus plasmids (41) yielded 99.0% and 99.6% length matches, with 755.8× and 591.1× average coverages and pairwise identities of 59.8% and 64.4%. Individual alignments to reference genomes are prone to reference induced bias. To assess the impact of reference bias, we simultaneously aligned Fresno16 to pX01/pBCX01 and pX02/pBC218. A slightly greater proportion of reads aligned to pBCX01 than pX01 (1.19:1), with similar average alignment scores (pX01, 9 [SD, 4]; pBCX01, 9 [SD, 3.78]); conversely, all reads for pX02/pBC218 preferentially mapped to pBC218.

Alignment of Fresno16 to pX01 plasmid toxin genes *pagA*, *lef*, and *cya* (42) yielded 81%, 100%, and 93.8% length matches at 14×, 478×, and 1,418× average coverage depths, respectively. Pairwise similarities of these matches were 53.9%, 87.1%, and 93.8%, respectively. Translation alignment of these three toxin genes showed at least one frameshift mutation and multiple coverage gaps for *pagA* and a frameshift mutation and a number of premature stop codons in *lef* and *cya*.

Alignment to the pX01 regulatory genes *atxA* and *pagR* (43, 44) yielded length matches of 100% for both at average coverage depths of 1,591.7× and 9.0×, respectively, with pairwise similarities of 55.1% and 76.0%, respectively. Translation alignment of the two genes yielded a frameshift mutation in *atxA* and 29 nonsynonymous single nucleotide polymorphisms (SNPs) in *pagR*. Alignment to the *capBCADE* operon of the pX02 plasmid (45, 46) yielded a 96.2% length match at an average coverage depth of 815.8× and a pairwise identity of 68.1%.

We did not find a significant correlation between human population size and number of AR genes using factorial logistic regression or analysis of covariance (ANCOVA) when controlling for species diversity (residual deviance = 0.10, degrees of freedom = 1, and *P* value = 0.74) and not controlling for species diversity (residual deviance = 5.85, degrees of freedom = 3, and *P* value = 0.12). ANCOVA results were similarly nonsignificant when species number was included (*F* value = 11.86 and *P* value = 0.18) and excluded (*F* value = 12.04 and *P* value = 0.17) as a covariate. We did not find significant correlation between the list of AR genes detected at each locality and the geographic distance between those localities (*R* = −0.27 and *P* value = 0.76) or significant correlation between the taxonomic composition of each locality and geographic distance between them (R = −0.41 and *P* value = 0.86), nor did we find a significant correlation between AR genes present and geographic distance when controlling for taxonomic composition (*R* = 0.10 and *P* value = 0.38) using full and partial Mantel tests for comparison. However, there was significant positive correlation between taxonomic composition and AR genes present (*R* = 0.80 and *P* value = 0.01).

## DISCUSSION

This study demonstrated a wide diversity of AR bacteria and AR genes present in wastewater samples in the Central Valley of California. Despite this region being a major agricultural production center (29), and a nexus for water transport from the Sierras to southern California (47), few studies of AR resistomes have focused on this region (48–51). Studies of geographic distribution of antibiotic resistance show varied results: a study of the freshwater lakes (52) and forest soils (53) showed patterns of variation by geographic distance over large spatial scales; conversely, another study of resistance genes present in glaciers did not find spatial structure, even at a global scale (54). Small-scale studies have shown similar variation in spatial distributions of catchment variation in antibiotic resistance genes, with variation detected in association with wastewater treatment plants (55) but not in association with agricultural runoff (56). Spatial patterns of antibiotic resistance fluctuate, and understanding the ecological and evolutionary dynamics of AR in the Central Valley is critical to developing predictions of AR impacts for the population of California as a whole.

Of the 16 genera and 18 species detected in this study, only 35% are routinely considered human pathogens (57); however, other isolates may cause infections in rare cases. This study therefore highlights that the evolution and spread of AR in the environment involves nonpathogenic vectors and reservoirs (23). Studies entirely focused on clinical isolates are insufficient to holistically understand the processes which govern antibiotic resistance (14, 15, 58).

The initial intent of this study was to specifically evaluate the population diversity of methicillin-resistant Staphylococcus aureus (MRSA) and carbapenem-resistant *Enterobacteriaceae*. However, none of the samples isolated from MRSA selective medium plates were S. aureus, and only 36% of the samples isolated from carbapenemase-producing *Enterobacteriaceae* plates were *Enterobacteriaceae*. While it should be acknowledged that the samples in this study were not clinical samples, it does raise the issue of potential misdiagnosis when using selective medium plates to identify clinical infections, a subject which has not been widely studied to date (59–61).

We found two isolates that displayed resistant phenotypes on selective media but did not detect any known resistance genes using genomic methods. There are two plausible explanations for these results; first, the sequencing effort may have not captured the resistance genes present in our data (62), and second, the resistance mechanisms in these bacteria may not be currently documented (63, 64). As both genera, *Paenibacillus* and *Weissella*, are not generally considered human pathogens (65, 66), it is possible that resistance mechanisms in these isolates require further characterization. However, the presence of these AR isolates in wastewater alongside significant human pathogens highlights their potential role as vectors of antibiotic resistance.

**FIG 7:**
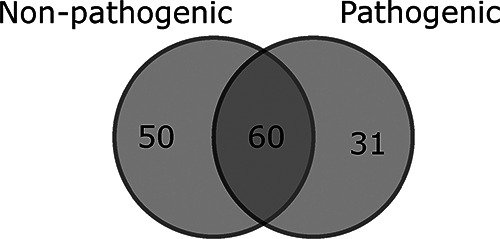
Venn diagram of antibiotic resistance genes specific to bacterial strains identified as pathogens and nonpathogens and those which were found in both groups. The largest proportions are shared, which suggests that nonpathogenic bacteria in wastewater can act as reservoirs of resistance that can be transferred to pathogens. The fact that many genes are also specific to each group highlights the necessity of considering ecological and evolutionary processes affecting both clinically relevant bacterial strains and the non-clinically relevant strains they may come into contact with in the broader environment in understanding the evolution and spread of antibiotic resistance.

The 100 genomes sequenced in this study were from both organisms known to be human pathogens and those not generally known to cause human infections (Fig. 7). Comparing the resistance genes present in pathogenic versus nonpathogenic species (57), it is observed that 35.5% of genes are specific to nonpathogens, 22.0% are specific to pathogens, and 42.6% are found in both nonpathogens and pathogens (Fig. 5). This analysis illustrates that horizontal gene transfer between pathogenic and nonpathogenic bacteria under diverse environmental conditions is likely to be a significant factor in the ecological and evolutionary dynamics of antibiotic resistance (23, 67). Evolutionary selection for antibiotic resistance in nonpathogenic bacteria can play a significant role in the development of resistant clinical infections (68). This result highlights that a comprehensive understanding of resistance mechanisms in the broader environment is necessary to provide context for studies focusing on antibiotic resistance in clinical settings. Trends observed in the clinic may be the result of processes occurring outside of it, potentially even in the case of nonpathogenic bacteria.

A large proportion of sequenced isolates (43.4%) had resistance genes on plasmids, indicating that conjugative transfer is an important mechanism in the development and spread of antibiotic resistance in wastewater communities (69, 70). Plasmids can be shared between distantly related bacterial species (71), further reinforcing the possibility of nonpathogenic bacterial populations contributing to clinically relevant antimicrobial resistance. Conversely, no resistance genes were identified in regions identified as prophage, despite 90% of isolates containing genomic regions identified as prophage sequences. This supports previous work that demonstrates that antibiotic resistance genes are rarely present in prophage (72, 73) and that conjugation is likely to be of greater importance in the evolution and spread of antibiotic resistance than transduction (74).

Multiple methods identify sample Fresno16 as Bacillus cereus; however, present in the genome of this sample were anthrax-like capsid and toxin plasmids. Other anthrax-like Bacillus cereus strains have been well characterized (41, 75), and the toxin-like plasmid in this strain is clearly distinct from previously described anthrax-like B. cereus, conversely capsid-like plasmid appears similar to other described capsid-like plasmids. Of note, B. anthracis and the anthrax-like B. cereus strains are generally not reported to be drug resistant (75, 76). We demonstrate that Fresno16 phenotypically demonstrates β-lactamase expression and contains genes encoding resistance to β-lactams (i.e., *bcI*, *bcII*, *bla1*, and *bla2*) (77, 78), fosfomycin (i.e., *fosB*) (79), mupirocin (*mupA*) (80), and vancomycin (i.e., *vanRM* and *vanZF*) (81, 82) and the multidrug resistance gene *vlmR* (83). This indicates that Fresno16 is likely multidrug resistant. Alignment of Fresno16 to anthrax pathogenicity genes suggests that it is unlikely that this strain is pathogenic in the same manner as anthrax and anthrax-like infectious agents, but this potential remains to be tested. It may be a worthy model for the study of multidrug-resistant, anthrax-like agents, pending further investigation of its phenotypic characteristics.

The correlation between species diversity and antibiotic resistance indicates that a population-scale evolutionary process may well be key to elucidating geographic patterns of antibiotic resistance (17, 58, 84). Factors affecting bacterial species diversity in the broader environment may be critical in predicting and managing environmental antibiotic resistance (14, 15, 67). We did not observe a significant correlation with geographic distance, although the geographic scale of the study may have been insufficient to detect regional variation in antibiotic resistance. It does raise the question of the landscape genetic processes which drive genetic interconnections between geographically discrete bacterial communities, which may be elucidated using population-level genetic studies (85).

## MATERIALS AND METHODS

### Sample collection, DNA extraction, and sequencing.

Single 1-day composite samples of wastewater influent from treatment plants in Fresno, Los Banos, Mariposa, Merced, and Modesto were collected in 50-ml Falcon tubes. Samples were transported on ice and stored at 2°C prior to being plated onto two ChromID (bioMérieux, France) selective medium plates—one MRSA (i.e., methicillin) and one CARBA (i.e., carbapenem) plate—according to manufacturer guidelines. Ten green-pigmented colonies from each plate (i.e., 20 total per site) were picked from each plate and cultured in liquid LB broth for 24 h. Whole genomic DNA from isolates was then extracted using an innuPREP bacterial DNA kit (Analytik Jena, Germany) according to the manufacturer’s guidelines, with the exception of the addition of lysozyme and lysostaphin to ensure complete lysis of cells. Library preparation was performed using an Illumina MiSeq V2 300-cycle kit in a paired-end configuration. Samples were pooled into two multiplexed libraries of 50 samples. Sequencing was performed at UC Merced using an Illumina MiSeq sequencer.

Sequences were quality filtered using Sickle v1.33 (86) using default settings (data not shown). Unassembled reads were taxonomically identified using both Kmerfinder 3 (87) and Strainseeker (88). Isolates were categorized as either pathogenic or nonpathogenic based on the NIH’s National Microbial Pathogen Data Resource (NMPDR) disease phenotype records and Public Health Agency of Canada’s pathogen safety data sheets (PSDSs). For *Bacillus* isolates, samples were compared to a custom BLAST database of diagnostic genes (for B. thuringiensis, Cry1 to Cry78; for B. cereus, Nhe, Hbl, and CytK; and for B. anthracis, pX01 and pX02) to diagnose species.

For sample Fresno16, we used BWA (89) to align trimmed reads to the Bacillus anthracis pX01 (GenBank accession no. CP008847.1) and pX02 (GenBank accession no. CP008848.1) plasmids and anthrax-like Bacillus cereus plasmids pBCX01 (GenBank accession no. NC_010934) and pBC218 (GenBank accession no. AAEK01000004) using default settings. Single nucleotide polymorphisms were called using the GATK pipeline (90). Consensus sequences of regions mapping to the toxin component genes (i.e., *pagA*, *lef*, and *cya*) and regulatory genes (i.e., *atxA* and *pagR*) of the pX01 plasmid, and the capsule synthesizer operon *capBCADE* of the pX02 plasmid, were translated and aligned to corresponding B. anthracis protein sequences using MUSCLE v3.8.31 (91) to determine the potential presence and functionality of these anthrax-specific genetic components. We also aligned the *plcR* gene to determine if Fresno16 carries this gene in either an activated or inactivated state. To assess reference bias in individual alignments, Fresno16 reads were simultaneously aligned to pX01/pBCX01 and pX02/pBC218 using GenomeMapper (92) using default settings.

*De novo* assembly of reads was performed using SPAdes v3.14.0 (93), and *de novo* plasmid assembly was performed using plasmidSPAdes v1.0 (94). Assemblies were compared to the Comprehensive Antibiotic Resistance Database (CARD) (95) using BLASTn (96). Matches to large, highly similar gene families [i.e., ACT, CMY, LEN, OKP, PDC, SHV, and TEM beta-lactamase families, ANT aminoglycoside modifiers, AAC(3) and AAC(6) acetyltransferases, MCR phosphoethanolamine transferase group, and quinolone resistance proteins] were considered single hits due to the sequence similarity of these groups. Prophage sequences for each isolate were estimated using PHASTER (97), and these were also compared to CARD (95) using BLASTn (96). Antibiotic resistance genes detected in both whole-genome assemblies and plasmid assemblies were visualized by site and by species using ggplot2 (98), and interactions between species and sites were visualized using upsetR (99).

In order to test the hypothesis that the diversity of AR genes is higher in larger urban centers, we determined if the human population size of the sampling locality was correlated with the number of resistance genes detected when controlling for species diversity and without, we conducted factorial logistic regressions using a Poisson linear model using the glm function in R (100) and ANCOVA using the aov function in R (101) to allow for the addition of controlling covariate data. Finally, to test the hypothesis that similarity of resistomes between sites was correlated with geographic proximity, we generated matrices of AR genes found at each site, and species diversity at each site using Jaccard/Tanimoto coefficients (102), and compared distance matrices using both full (i.e., AR genes × geographic distance, species diversity × geographic distance, and AR genes × species diversity) and partial (i.e., geographic distance × AR genes × species diversity) Mantel tests implemented in the Vegan package of R (103), using 9,999 permutations.

Whole genome sequencing data for this project is available through NCBI's Short Read Archive BioProject#PRJNA734303.
